# Rapidly Progressive Disseminated Intravascular Coagulation (DIC) in Severe Fatal Heatstroke: A Diagnostic Challenge Despite Normal Initial Coagulation Tests

**DOI:** 10.7759/cureus.81154

**Published:** 2025-03-25

**Authors:** Takahiro Tsuchida

**Affiliations:** 1 Department of Emergency Medicine, Obase Hospital, Fukuoka, JPN

**Keywords:** anticoagulation, coagulation disorder, critical care, disseminated intravascular coagulation, disseminated intravascular coagulation (dic), heat related, heatstroke, poor prognostic factor

## Abstract

This case report describes a fatal case of rapidly progressing disseminated intravascular coagulation (DIC) in a 50-year-old male with schizophrenia following severe classic (non-exertional) heatstroke. The patient, who was receiving antipsychotic medications (risperidone and olanzapine), presented with profound hyperthermia (41.7°C) and altered consciousness. Despite initial standard coagulation tests (prothrombin time (PT), activated partial thromboplastin time (APTT), and international normalized ratio (INR)) being within the normal range, overt DIC developed within three hours. This was characterized by a sharp decline in platelet count (from 28,000 to 6,000/µL), prolonged PT (from 12.6 to 39.2 seconds) and APTT (from 23.2 to 100.6 seconds), a marked increase in fibrin degradation products (FDP) (from 4.41 to 1,282 µg/mL), and fibrinogen depletion (from 339 mg/dL to below the measurement threshold), all consistent with overt DIC. The Japanese Association for Acute Medicine (JAAM) DIC score rapidly increased from 1 to 7. This deterioration coincided with the onset of acute kidney injury and hepatic dysfunction, supporting the hypothesis that heatstroke-induced coagulopathy has systemic effects. Despite aggressive treatment, including fluid resuscitation, extracorporeal cooling, vasopressors, blood product transfusion, antithrombin administration, and continuous hemofiltration, the patient succumbed to multi-organ failure 32 hours after admission. This case highlights the need for a high index of suspicion for DIC in severe heatstroke, even when initial coagulation tests appear normal. It also emphasizes the importance of early and continuous monitoring with more sensitive biomarkers, such as FDP, fibrinogen, and point-of-care viscoelastic testing (thromboelastography (TEG)/rotational thromboelastometry (ROTEM)). Early detection, rapid pre-hospital resuscitation, and targeted interventions are crucial to preventing progression to multi-organ failure. Future research should prioritize validating early diagnostic markers of heatstroke-induced DIC and developing specific therapeutic strategies.

## Introduction

Heatstroke is a life-threatening condition resulting from prolonged exposure to high temperatures, leading to systemic inflammatory response syndrome (SIRS) and multiple organ failure. Its global incidence is increasing, with mortality rates ranging from 10% to 50%, depending on severity [1,2]. As the most severe heat-related illness, heatstroke is defined by extreme hyperthermia (typically >40.5°C) and central nervous system dysfunction.

Heatstroke is classified as either classic (non-exertional) or exertional, depending on the underlying cause. Classic heatstroke typically affects elderly or chronically ill individuals with impaired thermoregulation, often occurring during heat waves. By contrast, exertional heatstroke occurs in healthy individuals engaging in strenuous physical activity in hot environments, where heat production exceeds the body's cooling capacity. Risk factors for classic heatstroke include extreme weather conditions, physiological vulnerabilities, social isolation, underlying illnesses, and medications that impair thermoregulation or cardiovascular function. Mortality rates exceed 50% in some populations [1,3]. Standard neuroprotective interventions in severe heatstroke include rapid cooling (cold water immersion, evaporative methods), sedation, neuromuscular blockade, seizure management, maintaining oxygenation, fluid resuscitation, and hemodynamic support to prevent secondary brain injury [4].

One of the most severe complications of heatstroke is disseminated intravascular coagulation (DIC), a life-threatening consumptive coagulopathy characterized by widespread microvascular thrombosis and subsequent hemorrhagic complications [5]. The pathogenesis of heatstroke-induced DIC is complex and involves widespread endothelial injury, excessive cytokine release, intestinal barrier dysfunction leading to endotoxemia, and ultimately, dysregulation of the coagulation cascade with concurrent fibrinolysis [5-7]. However, early detection of DIC remains challenging, as conventional coagulation parameters, namely, prothrombin time (PT), activated partial thromboplastin time (APTT), and international normalized ratio (INR), may initially remain within normal limits, delaying critical interventions. Although DIC is a well-established poor prognostic factor, standard coagulation tests may fail to detect abnormalities in its early stages, postponing potentially life-saving interventions [5,6].

This case report describes a patient with severe heatstroke who rapidly developed DIC despite initially normal coagulation parameters, highlighting the need for vigilant monitoring and more sensitive early biomarkers.

## Case presentation

A 50-year-old man with a history of schizophrenia, living alone, and receiving risperidone (2 mg/day) and olanzapine (20 mg/day), was found collapsed at home on a hot July day (maximum temperature: 33.8°C (92.8°F); humidity unknown). His hydration status before the collapse was unclear. He had no history of liver disease, anticoagulant or antiplatelet use, or drug abuse. He was initially brought to a local clinic, where he was diagnosed with grade II heatstroke and received intravenous fluids before being discharged home. He was reportedly able to walk upon returning home; however, no information was available regarding his vital signs or mental status at the clinic. His condition subsequently worsened, and approximately three hours later, he was found collapsed on the street and transported to our hospital.

Initial assessment at our hospital

Upon arrival, the patient was immediately assessed using the ABCDE approach. For airway management, endotracheal intubation was performed due to profoundly impaired consciousness. His respiratory rate was 22 breaths per minute, and oxygen saturation was 94% while receiving 15 L/min of oxygen via a non-rebreather mask. No abnormal respiratory sounds were noted. His blood pressure was 110/75 mmHg, and his pulse rate was 102 beats per minute. He was profusely diaphoretic with hot skin. Point-of-care echocardiography revealed an ejection fraction of 50% without regional wall motion abnormalities. The electrocardiogram showed no abnormalities. Fluid resuscitation with lactated Ringer’s solution was initiated immediately, with a total of 2,000 mL administered within the first three hours. The patient’s weight was 50 kg. Regarding neurological status, his Glasgow Coma Scale score was 6 (E1V1M4) upon arrival and remained at this level throughout the observation period. His pupils were 2+/2+ and reactive. No muscle rigidity was observed. For exposure and environmental control, his axillary temperature was 41.7°C, and his bladder temperature was 41.4°C. Given that he was found collapsed on a hot day, significant heat exposure was suspected, although the exact duration was unknown. Cooling measures, including ice packs and cooling blankets, were initiated immediately upon arrival. Notably, the patient’s body temperature decreased to 38.0°C within one hour of initiating cooling, meeting the critical "golden hour" target for heatstroke management [4]. Although additional methods such as evaporative cooling or ice water immersion were considered, they were not implemented due to the patient’s favorable initial response to ice packs and cooling blankets.

A whole-body computed tomography (CT) scan was performed. The head CT showed no evidence of intracranial hemorrhage. The thoracoabdominal CT revealed small bilateral pleural effusions and intestinal edema but no other significant findings. Initial coagulation tests, including PT, APTT, and INR, were within normal ranges (Table 1).

**Table 1:** Serial changes in coagulation tests: a dramatic FDP rise and fibrinogen depletion confirmed overt DIC. BUN: blood urea nitrogen; AST: aspartate aminotransferase; ALT: alanine aminotransferase; LDH: lactate dehydrogenase; CK: creatine kinase; CRP: C-reactive protein; Hb: hemoglobin; PT: prothrombin time; INR: international normalized ratio; APTT: activated partial thromboplastin time; FDP: fibrin degradation products; AT-Ⅲ: antithrombin III

	At arrival	Three hours after arrival	12 hours after arrival	Reference range
Sodium (mmol/L)	139	142	147	135-148
Potassium (mmol/L)	5.2	3.3	2.8	3.5-5.5
BUN (mg/dl)	38	39	44	7-24
Creatinine (mg/dl)	2.27	2.65	4.29	0.6-1.1
AST (U/l)	60	159	2034	13-30
ALT (U/l)	67	76	1608	3-42
LDH (U/I)	431	781	3183	115-245
CK (U/l)	1284	2415	8201	59-248
CRP (mg/dl)	0.44	0.3	0.17	0-0.3
White Blood Cell Count (/μl)	3,400	13,100	2,700	3,300-8,600
Hb (g/dl)	13.3	11.6	8.4	14-18
Platelet Count (/μl)	28,000	6,000	4,000	13,000-35,000
PT（sec）	12.6	39.2	38.7	9.8-12.4
INR	1.05	3.55	16.6	0.9-1.2
APTT（sec）	23.2	100.6	12.2	25.0-38.0
FDP (μg/ml)	4.41	1282	979.6	0-5.0
AT-Ⅲ (%)	97	58	79	70-120
Fibrinogen (mg/dl)	339	below the measurement threshold	70	200-400
D-dimer (μg/mL)	2.1	607.8	453.7	0.00-0.44
Lactate (mmol/L)	5	5.1		
Troponin I (ng/mL)	0.048			0-0.014

Clinical course

During the first hour after admission, cooling and supportive care were continued. Intravenous fluid administration led to blood pressure stabilization, and vasopressors were not required at this stage. Approximately three hours after admission, the patient developed hematemesis, melena, and gross hematuria. A rapid decline in platelet count (from 28,000/µL to 6,000/µL), along with prolonged PT (from 12.6 to 39.2 seconds) and APTT (from 23.2 to 100.6 seconds), strongly suggested consumptive coagulopathy. A marked elevation in fibrin degradation products (FDP) (from 4.41 to 1,282 µg/mL) and fibrinogen levels below the measurement threshold of the assay confirmed overt DIC. This was likely due to severe consumption secondary to DIC and the inherent limitations of the assay in detecting extremely low fibrinogen levels. The progression of the Japanese Association for Acute Medicine (JAAM) DIC score (from 1 to 7) further supported the diagnosis.

Treatment and outcome

While the elevated white blood cell (WBC) count and D-dimer levels could suggest sepsis as a differential diagnosis, the rapid onset of DIC following profound hyperthermia, the clinical context of a hot day, and negative culture results strongly indicated heatstroke-induced DIC as the primary cause. Elevated creatine kinase (CK) levels suggested heatstroke-associated rhabdomyolysis, likely contributing to the patient’s acute kidney injury. Despite aggressive, multimodal treatment-including platelet transfusions, fresh frozen plasma, antithrombin (36 units/kg, totaling 1,800 units), continuous renal replacement therapy (CRRT), and vasopressor therapy with noradrenaline-the patient’s condition continued to deteriorate. Although cryoprecipitate was considered a source of fibrinogen, it was unavailable at our institution at the time.

The patient ultimately developed circulatory shock, acute kidney injury, hepatic failure, and gastrointestinal bleeding, leading to death 32 hours after admission.

## Discussion

This case likely represents classic (non-exertional) heatstroke. Although the patient was receiving antipsychotic medications (risperidone and olanzapine), neuroleptic malignant syndrome (NMS) was considered unlikely due to the absence of muscle rigidity. While NMS can occur without rigidity, it was deemed less likely given the lack of significant autonomic instability, such as fluctuating blood pressure or persistent tachycardia, beyond what is expected in heatstroke [8]. In addition, although CK levels were elevated, they were consistent with heatstroke-induced rhabdomyolysis and not disproportionately high to suggest NMS. Antipsychotic drugs are known to impair thermoregulation by suppressing sweating and altering central temperature regulation, potentially increasing the patient’s susceptibility to heat stress, even in the absence of significant physical activity [8,9].

The JAAM DIC score, which considers underlying conditions (e.g., SIRS), platelet count, fibrinogen levels, FDP, and PT ratio, is commonly used to assess DIC severity. Higher scores correlate with an increased likelihood and severity of coagulopathy. Studies indicate that mortality rises with higher DIC scores, with a reported 10% mortality rate for a JAAM DIC score of 2 [10]. In another study, when D-dimer levels increased to more than five times the reference value, PT was prolonged by more than four seconds, and core body temperature reached ≥42°C, the mortality rate was 50% [11]. In this case, the patient’s coagulation function deteriorated rapidly within three hours. FDP increased sixfold, INR rose from 1.05 to 3.55, and fibrinogen levels decreased from 339 mg/dL to below the measurement threshold. The sharp decline in platelet count (from 28,000/μL to 6,000/μL) and the prolongation of PT (from 12.6 to 39.2 seconds) and APTT (from 23.2 to 100.6 seconds) strongly indicate consumptive coagulopathy. These changes confirm overt DIC and align with the rapid increase in the JAAM DIC score (from 1 to 7).

While standard coagulation tests (PT, APTT, and INR) may initially appear normal, more sensitive biomarkers can provide early indications of evolving DIC. Point-of-care viscoelastic tests, such as thromboelastography (TEG) and rotational thromboelastometry (ROTEM), offer a global assessment of coagulation, including clot formation, strength, and lysis. These tests can detect hyperfibrinolysis, a common feature of heatstroke-induced DIC, before significant PT/APTT changes occur [12]. In heatstroke-induced DIC, hyperfibrinolysis plays a complex and dynamic role, contributing to both hemorrhagic complications and microvascular thrombosis. Initially, excessive plasmin generation, often exceeding the inhibitory capacity of α2-plasmin inhibitor, leads to the degradation of fibrinogen and other coagulation factors, increasing the risk of bleeding. This early hyperfibrinolytic state is often followed by a suppressed fibrinolytic phase due to the delayed increase in plasminogen activator inhibitor-1 (PAI-1), which inhibits tissue-type plasminogen activator (t-PA) [12]. The FDPs generated during the hyperfibrinolytic phase can further interfere with fibrin polymerization and platelet function, contributing to the formation of microthrombi, particularly in the presence of endothelial damage caused by heat stress [11,12].

A prognostic scoring system for heatstroke has been proposed, incorporating temperature-related consciousness disturbances, liver dysfunction, renal dysfunction, and coagulation abnormalities [13]. However, research remains limited, and the level of evidence is low. Nevertheless, multiple studies have demonstrated a correlation between organ dysfunction severity and prognosis [4,13]. In this case, although coagulation abnormalities were initially absent, the patient presented with high fever, consciousness disturbances, and liver and renal dysfunction, suggesting a poor prognosis even before coagulation abnormalities appeared. This correlation indicates that the rapid onset of DIC is closely linked to acute kidney injury (AKI) and hepatic dysfunction, underscoring the systemic impact of heatstroke-induced coagulation disorders.

The management of severe heatstroke with DIC requires a multifaceted approach. Prompt and aggressive cooling, along with fluid resuscitation using crystalloid solutions, is essential to reduce core body temperature, treat dehydration, and maintain circulation. In addition, CRRT and therapeutic plasma exchange (TPE) may be beneficial for renal and hepatic dysfunction, though their use remains limited. Future research should explore early interventions to prevent multiple organ failure in such cases [3,4]. If DIC develops with clear coagulation abnormalities, blood products, such as platelet transfusions and fresh frozen plasma (FFP), may be required. The use of corticosteroids in severe heatstroke and DIC remains controversial [14]. In this case, corticosteroids were not administered due to the lack of evidence of adrenal insufficiency and concerns regarding potential immunosuppression, given the patient’s critical condition. The patient also received antithrombin therapy; however, the efficacy of antithrombin administration for heatstroke-induced DIC remains debated [6].

To improve the early diagnosis of DIC, clinicians should maintain a high index of suspicion in severe heatstroke cases, regardless of initial coagulation test results. Routine monitoring of sensitive markers, such as TEG and ROTEM, should be considered. Further research is needed to validate these markers and establish heatstroke-specific diagnostic thresholds.

## Conclusions

This case highlights the critical need for continuous and comprehensive coagulation monitoring in severe heatstroke patients, even when initial standard tests (PT, APTT, and INR) appear normal. The rapid progression to multiple organ failure, including AKI and hepatic dysfunction, underscores the systemic impact of heatstroke-induced DIC and the limitations of current standard treatments. Future research should prioritize the evaluation of early and sensitive biomarkers, such as FDP, fibrinogen levels, and point-of-care assessments using TEG or ROTEM, to enable earlier detection of DIC.

In addition, studies exploring potential therapeutic interventions, such as early plasma exchange or anticoagulation strategies tailored to the distinct pathophysiology of heatstroke-induced DIC, are essential. However, the efficacy of these interventions in heatstroke-induced DIC remains unestablished, warranting further investigation. This case underscores the need for novel treatment approaches, including proactive coagulation monitoring.

Crucially, rapid pre-hospital and in-hospital cooling, along with continuous monitoring, remain paramount in improving outcomes for patients with severe heatstroke.
